# The Dreadnought Hospital in the Royal Albert Dock

**Published:** 1903-08-01

**Authors:** 


### THE DREADNOUGHT HOSPITAL IN THE ROYAL ALBERT DOCK.

The immensity of London is always forcing itself upon the minds of the more thoughtful of its citizens. The portion of space which we denominate London, vast as it is, contains upon its surface in reality many cities, most of which differ from each other so widely as to make each of special interest to all who have eyes to see. Who can visit a place like Bow, and wander about amongst its houses and manufactories, without being struck by the beauty of its women, the healthiness and vitality of the children, and the amazing industry of every single inhabitant whom the stranger comes across in the course of his walk? It has been our good fortune to have to drive or walk through many parts of London during the past eight months, and in this way our eyes have been opened to the immense possibilities which all the varying circumstances of population and employment offer to the observant who will take the time to study the metropolis of the Empire as it deserves to be studied. Last week we found ourselves in East Greenwich, and then carefully inspected that splendid engineering feat the Blackwall Tunnel. As we came out on the Poplar side by the docks we found facing us these words: "If you are happy, remember the Poplar Hospital." This was not a quotation, nor was the name of the author appended, but everybody who knows anything about hospitals and those connected with them will have no difficulty in recognising its origin.

So we passed on and on through crowded narrow streets till we arrived at the Royal Albert Dock. Here, built upon piles, away from most things which men and women value in a place of residence, with no exercising ground for the resident and nursing staff save the docks and one lawn tennis court, stands the branch hospital of the old "Dreadnought" Society. We confess we were not prepared to find so well organised and so exceptionally well equipped an institution as this branch hospital undoubtedly is, with the School of Tropical Medicine attached to it. The large 20-bedded wards have been specially designed for clinical purposes, and the efforts of the architect have been seconded by the committee and medical staff, who have evidently spent much time and thought upon their equipment. Anyone furnishing a hospital would do well to go to the Royal Albert Docks Hospital and study especially the patients' lockers, the arrangements by which the prescription boards and medicine trays are attached to the walls, the hanging cupboards for mackintoshes and dressings, and the various glass-topped tables, with an excellent and simple arrangement whereby every piece of glass can be moved and cleansed. In fact this feature of ready adaptability in the furniture of the wards as securing absolute cleanliness proves in our judgment that someone connected with the institution, consciously or unconsciously, has here given an object-lesson in asepsis.

That object-lesson is being driven home, we fear at considerable cost to patients, in certain of our great hospitals, where tens of thousands of pounds spent upon new operation theatres have probably been practically wasted. This waste is caused by the failure to recognise that nothing matters really if only the individual surgeon is alert and alive to the fact that unless he is himself perfectly aseptic, and has his whole attention devoted to his own actions, his own instruments and his own work *vis-à-vis* with his patient, whatever else is done the patient may suffer injury, which may possibly result in death. In our view there must be a revolution before long in regard to the extravagant and unwarrantable expenditure which is at present going on in connection with these new operation theatres. It is time that science and commonsense cried "halt." It is time that the wise hint given by Mr. Watson Cheyne not very long ago in some pertinent but very guarded remarks should be taken to heart by every surgeon in active practice, and it is time, too, that the authorities of the great funds, and the responsible managers of every great hospital, should recognise that the present craze for extravagance and enormous outlay under certain items of expenditure in our hospitals, and especially upon the operation theatres, should be severely restrained with a view to reform.

The patients of this hospital present many features of interest, not only from the nature of the diseases from which they suffer, but on account of their nationality. Each ward with 20 beds contains representatives of many nationalities, and the habits and attitude of the patients to the trained observer convey much information. The hospital must be a most comfortable place of residence, for everything appears to be done to promote the well-being of the patients and also of the resident staff. It is a pity that the hospital and school have not a more extended site. We hope, however, it may be possible to arrange with the Dock Companies to take over a considerable portion of the ground now covered by storage sheds which are empty, and which appear to cumber the ground unnecessarily. In any case every advantage has been taken of the existing site, and the school buildings and large new mortuary present features of special interest, further mention of which must be left for another opportunity. We may, however, point out that the success and extent of the School of Tropical Medicine and the efficiency of its hospital seem to offer excellent reasons why representatives of both should be elected on the Central Hospital Council, if that body is to continue to exist for any purpose of public utility. The system of selecting students, the plan upon which instruction is given, the fees charged, and a multiplicity of other matters, based as they are upon modern methods, should make representatives of the School of Tropical Medicine a distinct addition to the Central Hospital Council.

